# PIV and CFD investigation of paddle flocculation hydrodynamics at low rotational speeds

**DOI:** 10.1038/s41598-022-23935-x

**Published:** 2022-11-17

**Authors:** Jean George Chatila, Hrair Razmig Danageuzian

**Affiliations:** Department of Civil Engineering, Lebanese American University, 309 Bassil Building, P.O.Box. 36, Byblos, Lebanon

**Keywords:** Environmental sciences, Engineering

## Abstract

In this study, flocculation hydrodynamics were evaluated by investigating the velocity field of turbulent flow experimentally and numerically, in a laboratory scale paddle flocculator. Turbulence that either promotes the particle aggregation or the breakage of flocs is complicated and has been considered and compared in this work using two turbulence models; namely, the SST k–ω and the IDDES. Results showed that IDDES provided very slight improvement as compared to SST k–ω, yielding the latter sufficient in accurately simulating the flow inside the paddle flocculator. A Goodness-of-Fit evaluation was adopted to study the convergence between PIV and CFD results, and to compare the results of the used CFD turbulence models. The study focused, as well, on the quantification of the slippage factor **k**, as 0.18 at low rotational speeds of 3 rpm and 4 rpm, and compared to conventional typical value of 0.25. This reduction of k from 0.25 to 0.18 yields around 27–30% increase in the power imparted to the fluid, and about 14% increase in velocity gradient (G). This implies that more mixing is provided than expected, and therefore, less energy is input and thus the electric consumption for the flocculation unit at a drinking water treatment plant could be potentially decreased.

## Introduction

In water treatment, the addition of coagulants destabilizes the fine colloidal particles and impurities, which then conjoin and form flocs in the flocculation stage. Flocs are loosely connected mass fractal aggregates, which are then removed by sedimentation. The characteristics of particles and the conditions of fluid mixing determine the action of flocculation and the efficiency of the treatment process. Flocculation requires slow mixing over relatively short periods, along with a high amount of energy to mix the large volume of water^1^.

In a flocculation process, the hydrodynamics of the entire system, in addition to the chemistry of the coagulant-particulate interactions determined the rate at which steady-state size distribution is achieved^2^. When particles collide, they would adhere to each other^3^. Oyegbile, Ay^4^ reported that collision is dependent on the flocculation transport mechanisms of Brownian diffusion, fluid shear, and differential settling. When flocs collide, they grow and reach beyond a certain limiting size and thus breakage might occur, as flocs do not endure the strength of the hydrodynamic forces^5^. Some of these broken flocs combine again to a lesser or same size^6^. However, strong flocs could endure the forces and maintain their size or even grow^7^. Yukselen and Gregory^8^ reported on research related to floc breakage and their ability to regrow, indicating that irreversibility was limited. Bridgeman, Jefferson^9^ used CFD to evaluate the local impact of mean flow and turbulence on floc formation and break-up using the local velocity gradient. In a basin provided with rotor paddles, it was necessary to change the speed of aggregate on collision with other particles when they were sufficiently destabilized in the coagulation phase^10^. Using CFD and at lower rotational speeds of around 15 rpm, Vadasarukkai and Gagnon^11^ were able to achieve the G-values used for tapered paddle flocculation, which minimized the power input required for mixing. However, operating at higher G-values may lead to floc breakup. They studied the influence of mixing speeds on the determination of the average velocity gradient for pilot-scale paddle flocculators. Their rotational speeds were greater than 5 rpm.

Korpijärvi, Ahlstedt^12^ used four different turbulence models to study the flow field of a jar test device. They used a laser-Doppler anemometer and PIV to measure the flow field and compared the calculated and measured results. de Oliveira and Donadel^13^ proposed an alternative approach for estimating the velocity gradient through hydrodynamic characteristics using CFD. The proposed approach was tested in six flocculation units based on helical geometry. The impact of retention time on flocculators was evaluated and a flocculation model was proposed, that can be used as a tool to support the rational design of low retention time units^14^. Zhan, You^15^ proposed a combined CFD model and the population balance model to simulate the flow characteristics and flocs behavior in a full-scale flocculation. Llano-Serna, Coral-Portillo^16^ investigated the flow characteristics of a Cox type hydraulic flocculator at the water purification plant in Viterbo, Colombia. Although applying CFD had its advantages, there were limitations such as numerical errors that existed in computations. Consequently, any numerical result obtained should be carefully examined and analyzed to make critical judgments^17^. Several studies focusing on the design of horizontally baffled flocculators existed in literature while design guidance for hydraulic flocculators has been limited^18^. Chen, Liao^19^ used an experimental setup based on polarized light scattering to measure the polarization states of the scattered light of the individual particles. Feng, Zhang^20^ simulated the vortex distribution and vorticity in the flow field of the same wave folded-plate flocculator and the opposite wave folded-plate flocculator by Ansys-Fluent. After using Ansys-Fluent to model turbulent fluid flow in hydraulic flocculators, Ghawi^21^ used the results in designing a hydraulic flocculator. Vaneli and Teixeira^22^ reported that there is still a lack of understanding of the relationship between the hydrodynamics of the helical tubular flocculators and the flocculation process, to support the rational design. de Oliveira and Costa Teixeira^23^ investigated the efficiency and presented a hydrodynamic characterization of helically coiled tube flocculators through physical experiments and CFD modeling. Helical tubes reactors or helically coiled tube flocculators were studied by many researchers. However, detailed fluid dynamics information on the response of these reactors to varying design and operational conditions is still lacking, (Sartori, Oliveira^24^; Oliveira, Teixeira^25^). Oliveira and Teixeira^26^ presented original results of theoretical, experimental and CFD modeling studies of helically coiled tube flocculators. Oliveira and Teixeira^27^ proposed using helically coiled tubes as a coagulation-flocculation reactor coupled with a conventional decanter system. They reported that the turbidity removal efficiency results obtained differ significantly from those obtained by the commonly used models for flocculation evaluation, which indicates caution in the use of such models. Moruzzi and de Oliveira^28^ performed simulations of system behavior of continuous flocculation chambers in series under different operating conditions, including variations in the number of chambers used and the utilization of fixed or scaled velocity gradients in the units. Romphophak, Le Men^29^ performed PIV measurements of instantaneous velocity in a quasi-two-dimensional jet clarifier. They detected the strong circulation induced by the jet in the flocculation zone and estimated the local and instantaneous shear rates.

Shah, Joshi^30^ reported that CFD provided an interesting alternative to improve a project and obtain a virtual response of flow. This helped avoiding the extensive experimental setups. CFD had been increasingly utilized to analyze water and wastewater treatment plants, (Melo, Freire^31^; Alalm, Nasr^32^; Bridgeman, Jefferson^9^; Samaras, Zouboulis^33^; Wang, Wu^34^; Zhang, Tejada-Martínez^35^). Some researchers conducted experiments on jar test equipment (Bridgeman, Jefferson^36^, Bridgeman, Jefferson^5^; Jarvis, Jefferson^6^; Wang, Wu^34^), and on perforated tray-type flocculators^31^. Others used CFD to evaluate hydraulic flocculators, (Bridgeman, Jefferson^5^; Vadasarukkai, Gagnon^37^). Ghawi^21^ reported that mechanical flocculators required regular maintenance as they suffered from continuous faults, and high amounts of electric energy.

The performance of paddle flocculators is highly influenced by basin hydrodynamics. An existing lack of the quantitative understanding of the velocity flow field in such flocculators is evidently identified in the literature, (Howe, Hand^38^; Hendricks^39^). The entire water mass is subjected to motion by the flocculator paddle wheel, and thus slippage is expected to occur. Normally, the velocity of the fluid is less than the velocity of the paddle by a slippage factor **k**, which is defined as the ratio of rotational velocity of water mass to rotational velocity of paddle wheel. Bhole^40^ reported that, in designing a flocculator, three unknown factors might be considered; namely, velocity gradient, drag coefficient, and relative velocity of the water with respect to the paddle.

Camp^41^ reported that when considering high-speed units, the velocity was about 24% of the speed of the rotors and reaches up to 32% for low-speed units. Without baffles, Droste and Gehr^42^ adopted a **k** value of 0.25, while with baffles **k** ranged between 0 and 0.15. Howe, Hand^38^ assumed k ranged between 0.2 and 0.3. Hendricks^39^ related the slippage factor with the rotational speed through an empirical formula and concluded that slippage factors also fell within the range established by Camp^41^. Bratby^43^ reported that **k** was about 0.2 for impeller speeds between 1.8 rpm to 5.4 rpm and increased to 0.35 at impeller speeds of 0.9–3 rpm. Other Researchers reported a wide range of values for the drag coefficient (C_d_) from 1.0 to 1.8 and the slippage factor **k** values of 0.25–0.40, (Fair and Geyer^44^; Hyde and Ludwig^45^; Harris, Kaufman^46^; van Duuren^47^; and Bratby and Marais^48^). Literature showed that no major advancements in the determination and quantification of **k** occurred after the work of Camp^41^.

The flocculation process relies on turbulence to promote collisions, where velocity gradient (G) is used to measure turbulence/flocculation. Mixing is the process whereby chemicals are quickly and uniformly dispersed in the water. The degree of mixing is measured by velocity gradient:

1 $$G=\sqrt{\frac{P}{\mu \forall }}$$

where G = velocity gradient (sec^−1^), P = power input (W), V = volume of water (m^3^), and μ = dynamic viscosity (Pa.s).

The higher the value of G the higher the mixing. Thorough mixing is essential if uniform coagulation is to occur. Literature indicates that the most important design parameters are mixing time (t) and velocity gradient (G). The flocculation process relies on turbulence to promote collisions, where velocity gradient (G) is used to measure turbulence / flocculation. The typical design values for G is 20 to 70 s^−1^, t is between 15 to 30 min, and Gt (dimensionless parameter) is between 10^4^ to 10^5^. Rapid mixing tanks operate best at G values from 700 to 1000, with detention times of approximately 2 min.

The power is defined by the following equation, ^42^:

2 $$P=\left(1.44 \times {10}^{-4}\right){ C}_{D} \rho b {\left[N\left(1-k\right)\right]}^{3} ({r}_{o}^{4}- {r}_{i}^{4})$$

where P is the power input imparted by each flocculator paddle blade to the fluid, N is the rotational speed, b is the blade length, ρ is the water density, r is the radius, and **k** is the slippage factor. This equation is applied individually to each paddle and then the results are summed to obtain the total power dissipation in the flocculator. A close examination of the equation shows the significance of the slippage factor **k** in the design process of paddle flocculator. The literature does not specify exact values for **k**, but rather ranges are recommended as explained previously. However, the relationship between the power P and the slippage factor **k** is cubical. Therefore, assuming all parameters the same, a variation of k from 0.25 to 0.3 for example yields around a 20% decrease in the power imparted to the fluid per paddle, while a decrease of **k** from 0.25 to 0.18 yields around 27–30% increase in the power imparted to the fluid per paddle. Ultimately, the impact of **k** on the sustainable design of paddle flocculators must be examined through its technical quantification.

Accurate empirical quantification of the slippage requires flow visualization and simulation. Thus, it was important to describe the water tangential velocities for certain rotational speed of the paddle at various radial distances from the shaft and at various depths from the water surface, thus evaluating the effect of the different positions of the paddle.

In this study, flocculation hydrodynamics was evaluated by investigating the velocity field of turbulent flow experimentally and numerically in a laboratory scale paddle flocculator. PIV measurements were recorded on the flocculator, which produced time-averaged velocity contours showing the velocity of water particles surrounding the blades. Also, CFD ANSYS-Fluent was used to model the rotational flow inside the flocculator, and time-averaged velocity contours were generated as well. The CFD models generated were validated, through a Goodness-of-Fit evaluation between the PIV and CFD results. The focus of this work concentrated on the quantification of the slippage factor k, which is a dimensionless parameter in the design of paddle flocculators. The work reported here provides a new basis for a quantitative determination of the slippage factor k at low rotational speeds of 3 rpm and 4 rpm. The impact of the study findings directly contributes to a better understanding of flocculator basin hydrodynamics.

## Materials and methods

### The laboratory scale paddle flocculator

**Figure 1:**
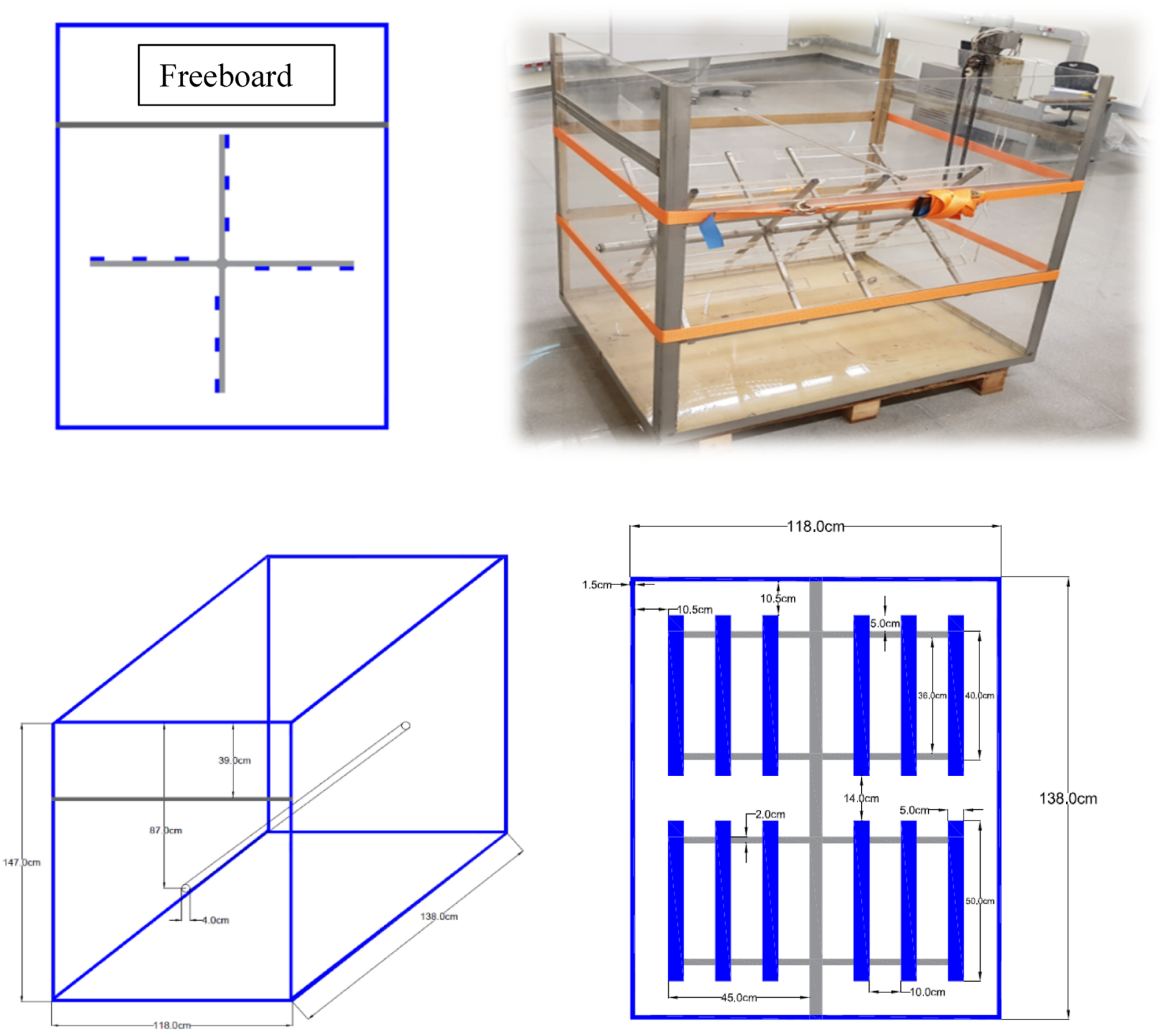
Schematic diagrams and basic dimensions of the experimental paddle flocculator.

The laboratory flocculator constituted a rectangular box with a top opening, with a total height of 147 cm with a 39 cm freeboard, a total width of 118 cm, and a total length of 138 cm, (Fig. 1). The basic design criteria developed by Camp^49^ were adopted in designing a laboratory scale paddle flocculator along with applying the principles of dimensional analysis. The experimental setup was built in the Environmental Engineering Laboratory at the Lebanese American University (Byblos, Lebanon).

A horizontal shaft is situated at a height of 60 cm from the bottom that holds two paddle wheels. Each paddle wheel consisted of 4 arms, and each arm held three blades, making a total of 12 blades. Flocculation required gentle mixing at low rotational speeds ranging between 2 to 6 rpm. The most common rotational speeds for mixing in flocculators were 3 rpm and 4 rpm. The flow inside the laboratory scale flocculator was designed to represent flow inside a compartment of a flocculation basin at a drinking water treatment plant. The power was computed using the traditional equation^42^. For both rotating speeds, the velocity gradient $\stackrel{\mathrm{-}}{\text{G}}$ values were above 10 ${\text{sec}}^{-{1}}$, and the Reynolds’ Numbers indicated turbulent flow, (Table 1).

**Table 1:** Power, $\stackrel{\mathrm{-}}{\text{G}}$ and Reynolds numbers for the two rotating speeds.

	3 rpm	4 rpm
P (watts)	0.2	0.5
$\stackrel{\mathrm{-}}{\text{G}}$ (${\text{s}}^{-{1}}$)	11	17
Re	44,180	58,910

### PIV experimental setup and procedure

**Figure 2:**
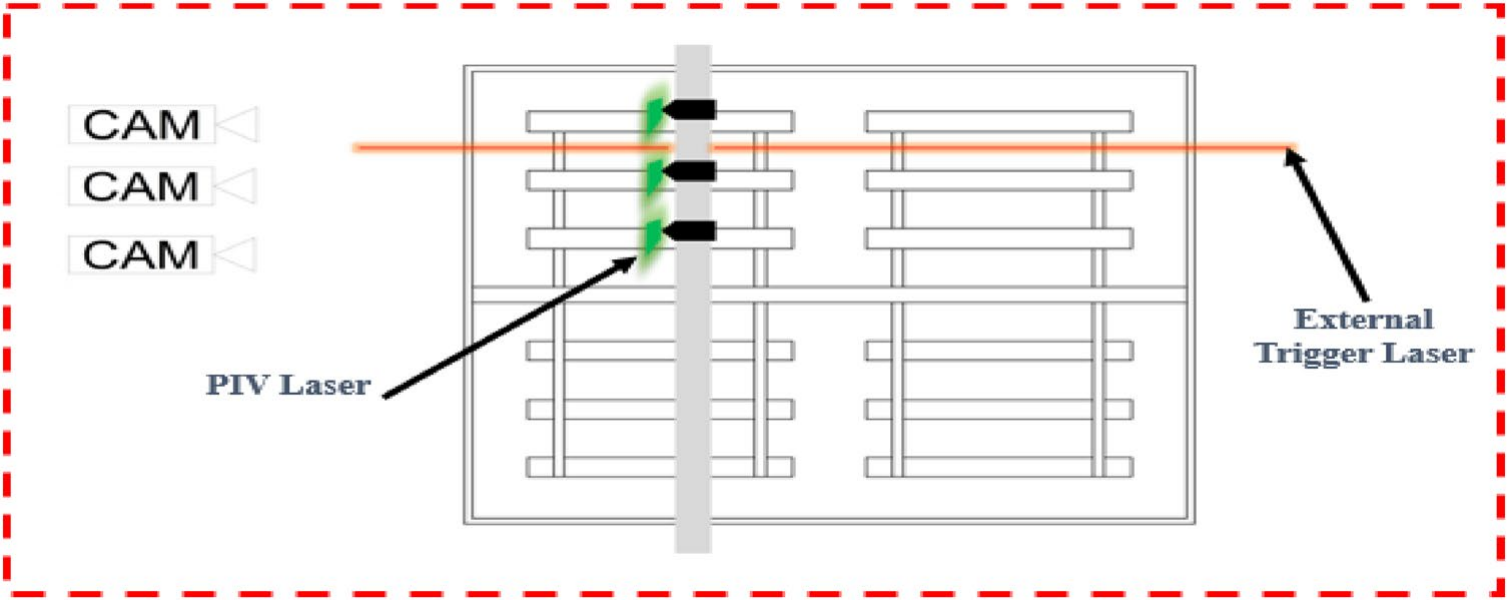
PIV setup, image acquisition process and location of recorded images.

PIV was used to achieve accurate and quantitative measurement of fluid velocity vectors at a very large number of points simultaneously^50^. The experimental setup consisted of the laboratory scale paddle flocculator, the LaVision PIV system (2017), and an Arduino laser-sensor external trigger. To produce time-averaged velocity contours, PIV images were recorded consecutively at the same location. The PIV system was calibrated such that the target area was at mid-length of each of the three blades of a particular arm of the paddle wheel. The external trigger consisted of a laser that was placed on one side of the flocculator width, and a sensor receiver that was situated on the other side. Every time the flocculator arm obstructs the path of the laser, a signal was sent to the PIV system to capture an image through the PIV laser and camera that were synchronized using the programmable timing unit. Figure 2 shows the setup of the PIV system and the image acquisition process.

PIV recordings started after the flocculator has operated for 5–10 min to normalize the flow and to account for the same refractive index field. Calibration was achieved using a calibration plate that was submerged in the flocculator and placed at mid-length of the blade of interest. The PIV laser location was adjusted to form a planar light sheet exactly above the calibration plate. Measurements were recorded per blade per rotational speed, and the selected rotational speeds for the experiments were 3 rpm and 4 rpm.

For all the PIV recordings, time separation between two laser pulses was adjusted between 6900 and 7700 μs by allowing a minimum particle shift of 5 pixels. Trial tests concerning the number of images needed to get accurate time-averaged measurements were performed. Vector statistics of samples containing 40, 50, 60, 80, 100, 120, 160, 200, 240 and 280 images were compared. It was found that a sample size of 240 images produce a stable time-averaged result noting that each image consisted of two frames.

Since the flow was turbulent in the flocculator, small interrogation window sizes were required with a high number of particles to resolve the small turbulent flow structures. Decreasing size multi-pass iterations were applied with the cross-correlation algorithm to ensure accuracy. An initial interrogation window size of 48 × 48 pixels with a 50% overlap and one adaptation pass followed by a final interrogation window size of 32 × 32 pixels with a 100% overlap and two adaption passes were applied. Furthermore, glass hollow spheres were used as seeding particles in the flow whereby a minimum of 10 particles were allowed per interrogation window. A PIV recording was initiated with a trigger source inside the programmable timing unit (PTU) which was responsible for the operation and synchronization of the laser light source and the camera.

### Computational fluid dynamics (CFD) flow simulation

The commercial CFD software package ANSYS Fluent v 19.1 was used to develop the 3D models and to solve the governing flow equations.

#### Geometry setup

**Figure 3:**
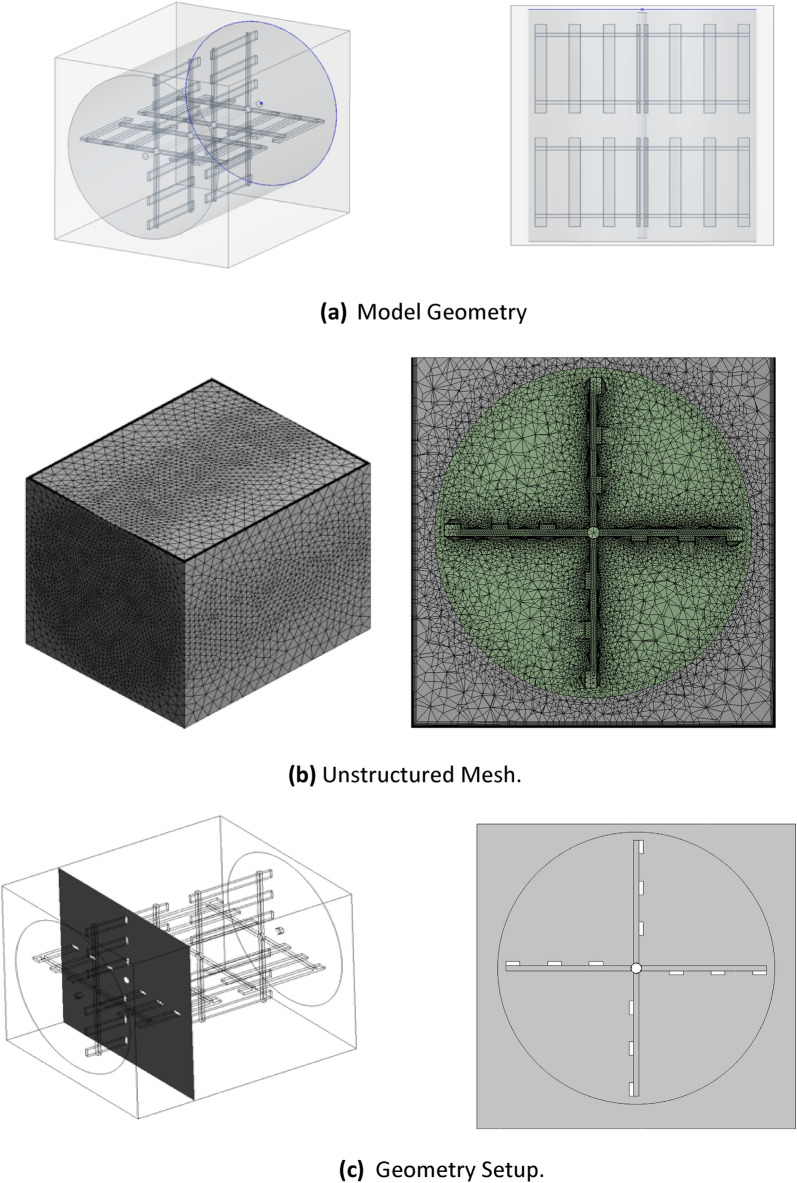
ANSYS-fluent 3-D and schematic views of model geometry, ANSYS-fluent mesh of flocculator body at plane of interest, ANSYS-fluent schematic views at plane of interest.

A 3D model was generated of the laboratory scale paddle flocculator using ANSYS-Fluent. The model was created as a rectangular box consisting of two paddle wheels fixed on a horizontal shaft identical to the laboratory model. The model height was 108 cm excluding the freeboard, with a width of 118 cm, and a length of 138 cm. A horizontal cylindrical-shaped plane was added encompassing the mixer. The generation of the cylindrical plane was needed to achieve rotation of the entire mixer in the setup phase, and to simulate the rotational flow field inside the flocculator as shown in Fig. 3a.

The model geometry consisted of two domains, where both were fluids. This was achieved by using the Boolean subtract function. Initially the cylinder (including the mixer) was subtracted from the box to represent the fluid. Then, the mixer was subtracted from the cylinder thus yielding two bodies, the mixer and the fluid. Finally, a sliding interface was applied between the two domains, namely a box-cylinder interface and a cylinder-mixer interface, (Fig. 3a).

#### Mesh generation

Mesh generation for the built model was completed to fit the requirements of the turbulence models, which would be used in running the numerical simulations. An unstructured mesh with inflation layers near solid surfaces was utilized. Inflation layers were created for all walls, with a growth rate of 1.2 ensuring that complex flow structures were captured, and a first layer thickness of $7\mathrm{ x }{10}^{-4}$ m ensuring that ${\text{y}}^{+}\le 1.0$. Body sizing through a patch conforming method using tetrahedrons was applied. Face sizing of the two interfaces with an element size of 2.5 × ${10}^{-3}$ m was generated, while face sizing of the mixer originated with an element size of 9 × ${10}^{-3}$ m was applied. The original generated mesh was composed of 2,144,409 elements, (Fig. 3b).

#### Turbulence models

The two-equation k–ε turbulence model was selected as a starting baseline model. Computationally more expensive models were selected to accurately simulate the rotational flow inside the flocculator. Two CFD models were adopted to numerically study the turbulent rotational flow inside the flocculator; namely, the SST k–ω^51^ and the IDDES^52^. Results from both models were compared with the PIV experimental results for model validation. Firstly, the SST k–ω turbulence model is a two-equation eddy-viscosity model that is used for hydrodynamic applications. It is a hybrid model combining the Wilcox k-ω and the k-ε models. A blending function activates the Wilcox model near the wall and the k-ε model in the free stream. This ensures that the appropriate model is utilized throughout the flow field. It accurately predicts flow separation due to adverse pressure gradients. Secondly, the widely used the Improved Delayed Detached Eddy Simulation (IDDES) method of the Detached Eddy Simulation (DES) model with a SST k–ω RANS (Reynolds Averaged Navier Stokes) model was selected. IDDES is a hybrid RANS-LES (Large Eddy Simulation) model that provides a more flexible and convenient Scale Resolving Simulation (SRS) model. It relies on the LES models to resolve the large eddies and reverts to SST k–ω to model the small-scale eddies. Statistical analysis of results obtained from simulations using SST k–ω and IDDES were compared to PIV results for model validation.

#### Turbulence models

The two-equation k–ε turbulence model was selected as a starting baseline model. Computationally more expensive models were selected to accurately simulate the rotational flow inside the flocculator. Two CFD models were adopted to numerically study the turbulent rotational flow inside the flocculator; namely, the SST k–ω^51^and the IDDES^52^. Results from both models were compared with the PIV experimental results for model validation. Firstly, the SST k–ω turbulence model is a two-equation eddy-viscosity model that is used for hydrodynamic applications. It is a hybrid model combining the Wilcox k-ω and the k-ε models. A blending function activates the Wilcox model near the wall and the k-ε model in the free stream. This ensures that the appropriate model is utilized throughout the flow field. It accurately predicts flow separation due to adverse pressure gradients. Secondly, the widely used the Improved Delayed Detached Eddy Simulation (IDDES) method of the Detached Eddy Simulation (DES) model with a SST k–ω RANS (Reynolds Averaged Navier Stokes) model was selected. IDDES is a hybrid RANS-LES (Large Eddy Simulation) model that provides a more flexible and convenient Scale Resolving Simulation (SRS) model. It relies on the LES models to resolve the large eddies and reverts to SST k–ω to model the small-scale eddies. Statistical analysis of results obtained from simulations using SST k–ω and IDDES were compared to PIV results for model validation.

#### ANSYS-fluent setup

A pressure based and transient solver was employed, and using the gravitational acceleration in the Y-direction. Rotation was achieved by assigning a mesh motion to the mixer, where the rotational axis origin was the center of the horizontal shaft and the rotation axis direction was in the Z. A mesh interface was created for the two interfaces of the model geometry, producing the two boundary zone sides. Similar to the experimental procedure, the equivalent of a rotational speed of 3 rpm and 4 rpm.

Boundary conditions for the mixer and the flocculator walls were assigned as walls, while the top opening of the flocculator was assigned as an outlet vent with a gauge pressure equal to zero, (Fig. 3c). The SIMPLE scheme was used for the pressure–velocity coupling, and least squares cell based for the spatial discretization of the gradient with second order functions for all parameters. The convergence criterion of all flow variables was a scaled residual of 1 x ${10}^{-3}$. The maximum number of iterations per time step was 20, with a time step size equivalent to 0.5° rotation. The solution converged at the 8th iteration for the SST k–ω model and at the 12th iteration using IDDES. Also, the number of time steps was calculated such that the mixer would rotate a minimum of 12 complete rotations. Data sampling for time statistics was applied after 3 rotations allowing the flow to normalize similar to the experimental procedure. Comparison of velocity contours exported at each rotation yielded precisely identical results for the last four rotations indicating that steady state was achieved. Additional revolutions did not improve the averaged velocity contouring.

#### Time step size and mesh refinement

Determination of the time step size was related to the rotational speed as 3 rpm or 4 rpm. The time step size was refined to the time needed for the mixer to rotate 0.5°. This showed to be adequate since the solution readily converges, as described in the aforementioned section. Therefore, all numerical simulations for both turbulence models were conducted using the modified time step size of 0.02 $\stackrel{\mathrm{-}}{7}$ for 3 rpm, and 0.0208 $\stackrel{\mathrm{-}}{3}$ for 4 rpm. The cell Courant Number was always less than 1.0 using the refined time step sizes stated.

In an attempt to study the dependency of model on the mesh, results were generated first using the original mesh having 2.14 million elements followed by the refined mesh having 2.88 million elements. Mesh refinement was achieved by decreasing the element size of the mixer body from 9 × ${10}^{-3}$ m to 7 × ${10}^{-3}$ m. Average values of velocity magnitude at different locations surrounding the blades were compared for the original and refined mesh of both turbulence models. The percent difference between the results was 1.73% for the SST k–ω model and 3.51% for the IDDES model. IDDES showed a higher percent difference since it is a hybrid RANS-LES model. These differences were considered negligible, and therefore the original mesh having 2.14 million elements with a time step size of 0.5° rotation was used to perform the simulations.

## Results and discussion

### Accuracy and precision of PIV results

The reproducibility of experimental results was studied by conducting each of the six experiments a second time and comparing results. The velocity values at the center of blades were compared for the two sets of experiments. The average percentage difference between the two experimental sets was 3.1%. The PIV system was also re-calibrated independently for each experiment. Analytically computed velocity at the center of each blade was compared with the PIV velocities at the same location. This comparison showed discrepancies, with a maximum percent error of 6.5% at blade 1.

### Analysis of PIV results

The quantification of the slippage factor must be preceded by a scientific understanding of the concept of slippage in paddle flocculators, hence there is a need for examining the flow structures around the flocculator paddle blades. Conceptually, the slippage factor is incorporated in the design of paddle flocculators to account for the speed of the blades relative to that of water. Literature proposes this speed to be 75% of the blade speed, hence k is commonly adopted as 0.25 in most designs to account for this adjustment. To this end, velocity streamlines generated experimentally using PIV are needed to understand fully the velocity flow field and investigate this slippage. Blade 1 was the innermost blade closest to shaft and Blade 3 was the outermost one, while blade 2 was the middle one.

#### Average velocity streamlines at blade 1

**Figure 4:**
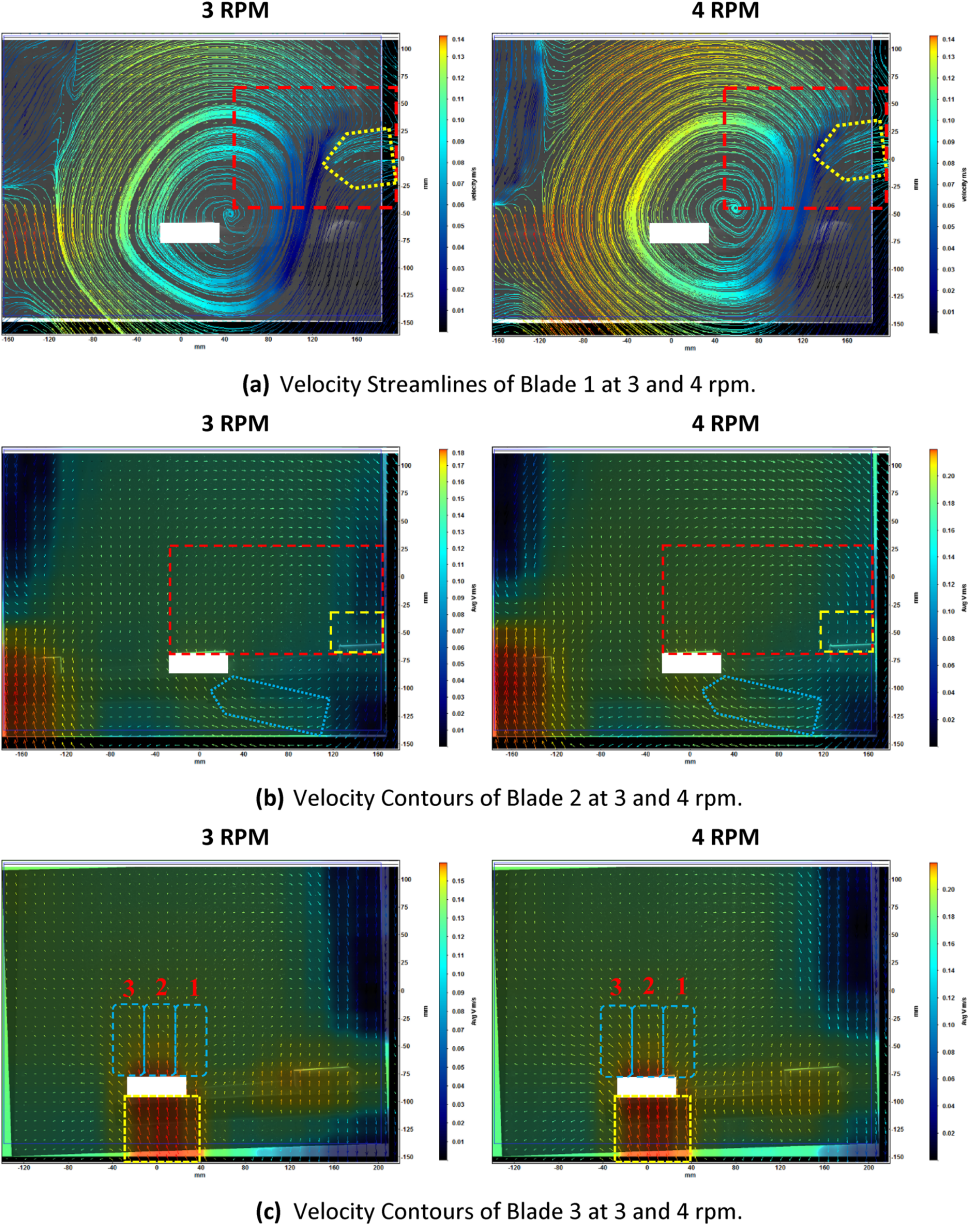
Velocity fields and streamlines of the three blades at the two rotating speeds of 3 and 4 rpm. Maximum average velocity at 3 rpm was 0.15 m/s, 0.20 m/s and 0.16 m/s respectively for the three blades, and the maximum average velocity at 4 rpm was 0.15 m/s, 0.22 m/s and 0.22 m/s respectively for the three blades.

Velocity streamlines at blade 1 showed a direct rotational flow surrounding and encompassing the blade. These flow structures stemmed from a point located to the right of the blade, which was between the rotor and the blade. Inspecting the area denoted by the dashed red box in Fig. 4a, it was interesting to identify another aspect of the re-circulating flow over and around the blade. Flow visualization showed a small flux joining into the re-circulation zone. This flux was approaching from the right side of the blade and at a height of about 6 cm from the blade face, which would be due to the impact of the first blade of the arm preceding the one seen in the image. Flow visualization at a rotational speed of 4 rpm showed the same behavior and structures, evidently with higher velocity values.

#### Average velocity streamlines at blade 2

Another form of spiral flow is identified between blades 1 and 2. The vector field visibly indicated flow of water from beneath blade 2 moving upwards as noted by the vector directions. As seen in the dashed box in Fig. 4b, these vectors were not proceeding vertically upwards from the face of the blade, but rotating towards the right and gradually downwards. Downward directed vectors were identified on the face of blade 1 approaching from the re-circulating flow that formed between these two blades and encompassed both. The same flow structures were identified for both rotational speeds with higher velocity magnitude at 4 rpm.

#### Average velocity streamlines at blade 3

The velocity field of blade 3 did not show a major contribution of velocity vectors from previous blades that were joining the flow beneath blade 3. The main flow flux beneath blade 3 was coming from vertical velocity vectors directed upwards with the flow of water.

The velocity vectors above the face of blade 3 could be divided into three sets, as shown in Fig. 4c. The first set was the one at the right periphery of the blade. Flow structures at this location were directly rotating upwards to the right (i.e. towards blade 2). The second set was considered at the middle of the blade. Velocity vectors at this location proceeded vertically upwards without any deviation, and did not show any rotation. A decrease in velocity magnitude was identified with increasing height above the blade face. For the third set, which was at the left periphery of the blade, flow was directed immediately to the left, i.e. towards flocculator walls. The majority of the flow, represented by the velocity vectors, proceeded upwards, while part of the flow advanced horizontally downwards.

### Analysis of ANSYS-fluent results

**Figure 5:**
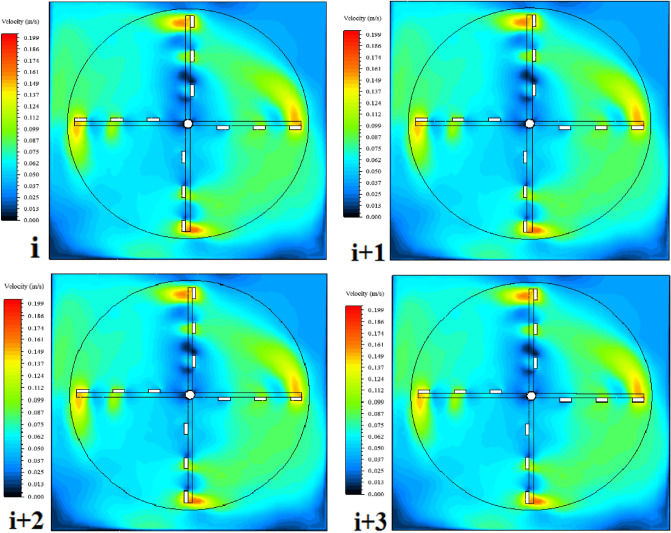
Similarity between CFD velocity contours generated at four consecutive rotations.

**Figure 6:**
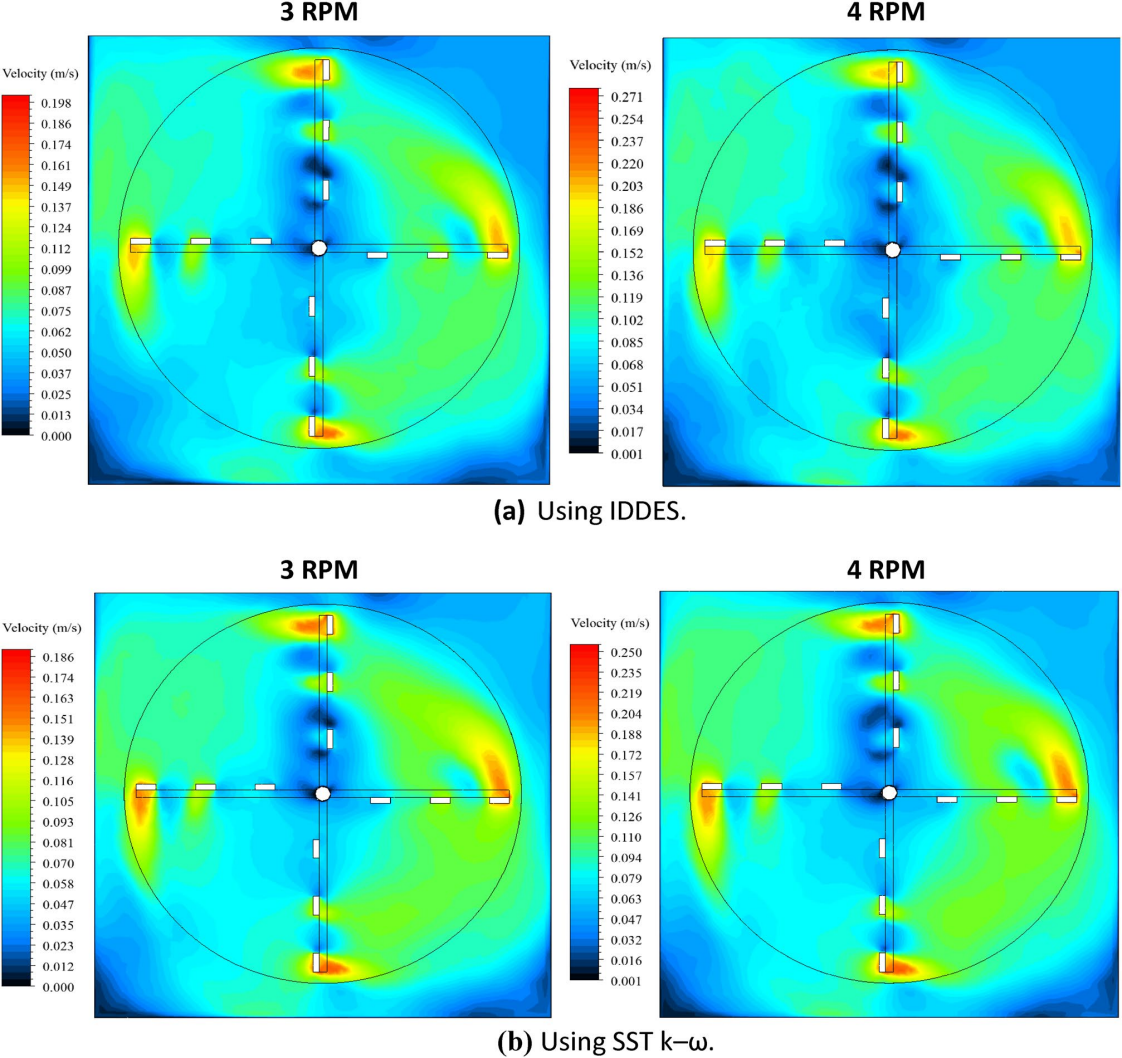
Time-averaged velocity contours generated using IDDES and SST k–ω with velocity contour scales higher for IDDES.

Time-averaged velocity contours were generated using both turbulence models, SST k–ω and IDDES, for both rotational speeds of 3 rpm and 4 rpm at a plane situated at mid-length of blades. Steady state was achieved by realizing absolute similarity between the velocity contours generated at four consecutive rotations as seen in Fig. 5. Also, time-averaged velocity contours generated using IDDES are shown in Fig. 6a while time-averaged velocity contours generated using SST k–ω are shown in Fig. 6b.

**Figure 7:**
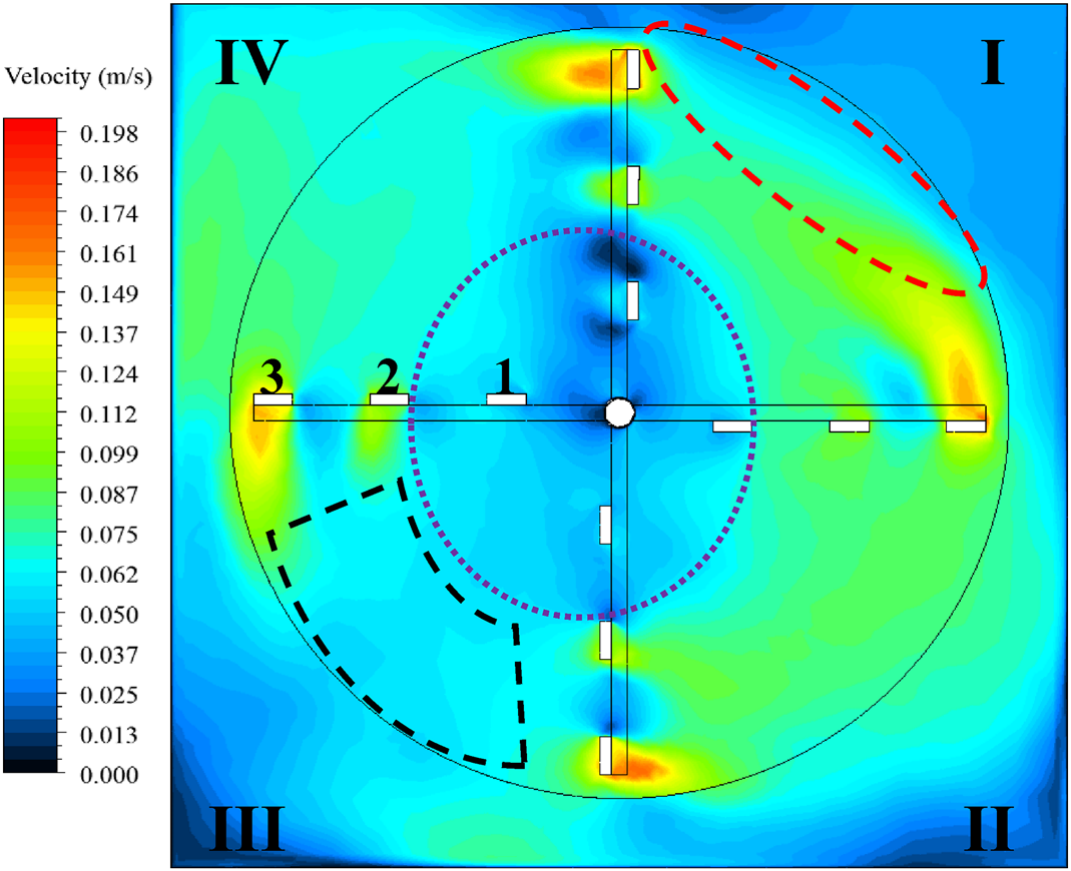
Close inspection of velocity contour generated using IDDES at 3 rpm.

A close inspection of the velocity contour generated by using IDDES at 3 rpm was considered and shown in Fig. 7. The mixer is rotating clockwise, and the flow was discussed according to the annotations shown.

Figure 7 shows flow separation was identified at the face of blade 3 in quadrant I since the flow was not bounded due to the presence of the top opening. In quadrant II, no flow separation was seen since the flow was completely bounded by the flocculator walls. While in quadrant III, water rotated at noticeably low or lower velocity magnitudes as compared to the previous quadrants. Water was being moved (i.e. rotated or pushed) downwards by the impact of the mixer in quadrants I and II. Whereas in quadrant III, water was being pushed upwards by the arm of the mixer. Clearly, there was resistance from the water mass at this location onto the approaching flocculator arm. There was complete detachment of the rotational flow in this quadrant. Regarding quadrant IV, a large part of the flow above blade 3 was directed towards the flocculator wall, and this flow gradually lost its magnitude with increasing height up to the top opening.

Furthermore, as denoted by the dashed oval in blue, the location at the center includes complex flow structures, which were predominant in quadrants III and IV. This denoted area did not show any association with the rotational flow in the paddle flocculator, as swirling motion could be identified. This was in contrast to quadrants I and II, where there was a clear separation between this inner flow and the total rotational flow.

As shown in Fig. 6 and comparing the results from IDDES and SST k–ω, the main difference in the velocity contours was the velocity magnitude right beneath blade 3. The SST k–ω model clearly showed an extended high velocity flow being carried by blade 3 as compared to IDDES.

Another difference can be identified in quadrant III. From IDDES, rotational flow detachment between the flocculator arms was noticed as noted earlier. However, this location was greatly impacted by flow with low velocity magnitude joining from the corner and from the inner flow of the first blades. From SST k–ω, and for the same location, the contours showed relatively higher velocity magnitude as compared to IDDES since there were no joining flows from other regions.

### Velocity profile plots

**Figure 8:**
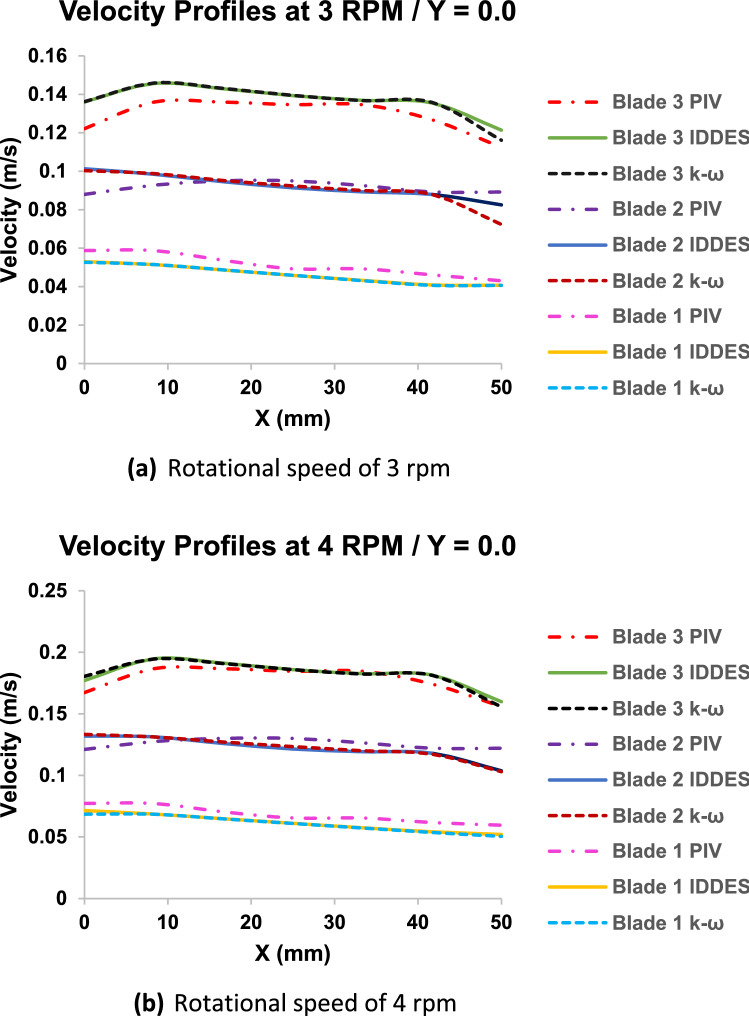
PIV, IDDES and SST k–ω velocity profiles at the surface of the blade. The x-axis represents the width of each blade in mm, with the origin (0 mm) represents the left periphery of blades, while the end (50 mm) represents the right periphery of blades.

A qualitative understanding of the velocity vector fields and streamlines is required to have a proper understanding of flow behavior and structure. Noting that the width of each blade is 5 cm, 7 velocity points were selected across the width ensuring the production of representative velocity profiles. Also, a quantitative understanding of the variation of velocity magnitude with increasing height above blade faces was required through plotting velocity profiles right above at the face of each of the blades, and at successive distances of 2.5 cm vertically upwards up to a height of 10 cm. For more details refer to Figs. S1, S2 and S3 in Appendix A. Figure 8 showed the similarity of the velocity profiles at the surface of each of the blades (Y = 0.0) generated using PIV experiments and ANSYS-Fluent analyses using IDDES and SST k-ω. Both numerical models were accurate in simulating the flow structures at the surface of flocculator blades.

It can be clearly identified that the velocity profiles of blades 2 and 3, as shown in Fig. 8 and Figs. S2, S3 in Appendix A, showed a trend of similarity with increasing height, while that of blade 1 varied independently. Velocity profiles of blades 2 and 3 became completely straight and at equal magnitude at a height of 10 cm from blade faces. This implied that the flow became uniform at this location. This was most clearly noted from the PIV results, which were closely replicated by IDDES. While, the SST k–ω results showed some variations particularly at 4 rpm.

It is important to note that blade 1 maintained the same velocity profile shape at all positions and did not normalize with height because of the swirling flow formed at the center of the mixer encompassing the first blade of all arms. Moreover, velocity profiles for blades 2 and 3 from PIV showed slightly higher velocity magnitudes at most positions as compared to IDDES, until becoming almost equal at a height of 10 cm above blade face.

### Model validation

**Figure 9:**
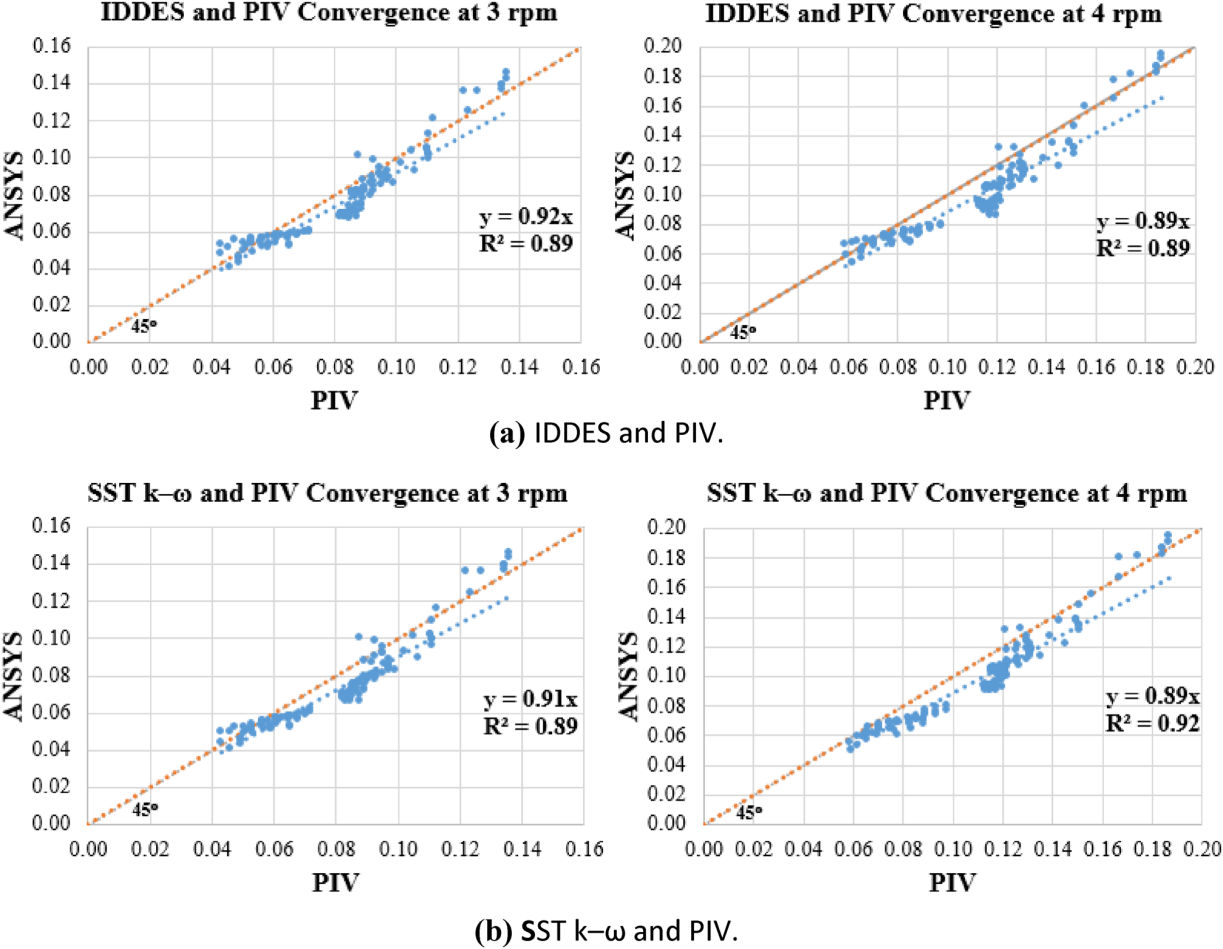
Goodness-of-fit evaluation of computed velocities and PIV measurements.

A Goodness-of-Fit Evaluation was adopted to study the convergence between PIV and CFD results and compared the results of the CFD turbulence models used, (Fig. 9). The Goodness-of-Fit evaluation is based on the coefficient of determination ${\text{r}}^{2}$, and one specific linear correlation should be taken into consideration: ${\text{Y}}_{\text{observed}} \, = \text{ 1 } \times \, {\text{Y}}_\text{predicted }+ \text{ 0}$, i.e. the 1:1 line.

The results of the Goodness-of-Fit evaluation, as shown in Fig. 9, showed that the coefficient of determination is almost equal to 0.9 and greater for all comparisons. The slopes of the best-fit lines were also approximately 0.9 and greater, indicating good agreement between the PIV and CFD results.

**Figure 10:**
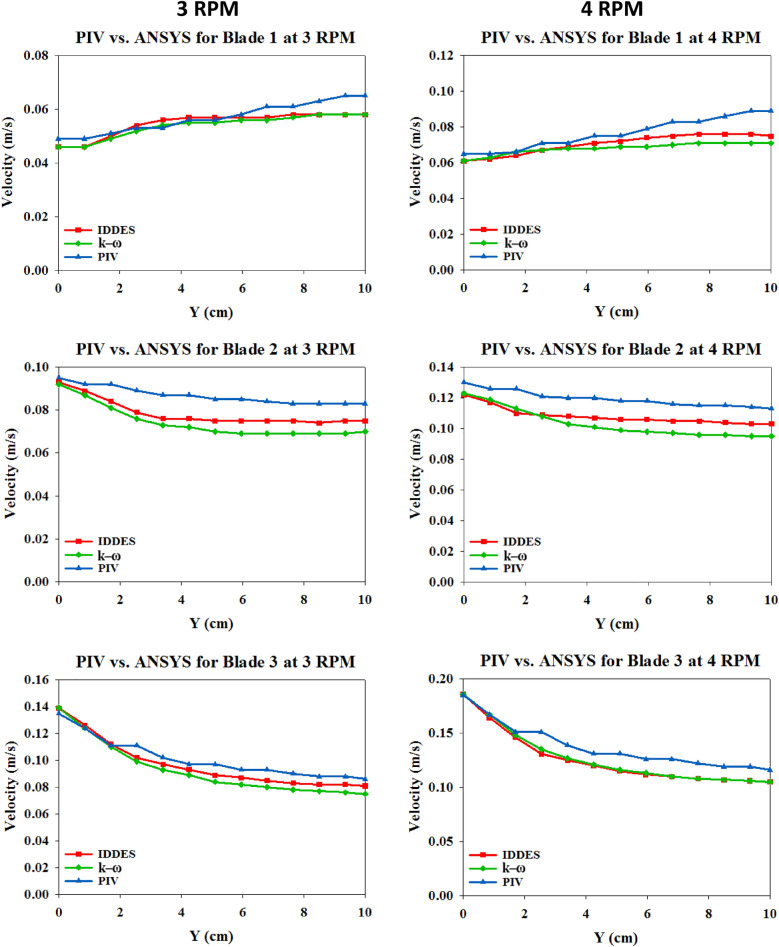
Comparison of PIV and ANSYS-fluent velocity variation above blade faces.

In addition, the variation of velocity magnitude above the face of each of the blades was considered, where velocity magnitude was extracted at thirteen points starting from the center of the face of each blade and up to a height of 10 cm above blade face as seen in Fig. 10.

The x-axis in Fig. 10 represented the height above blade face starting from the origin which was at the face of the blade and upwards up to Y = 10 cm. The y-axis represented the velocity of the water particles in m/s. Regarding blade 1, the velocity of water particles was increasing with height above the blade face for both rotational speeds. In general, this was unpredictable since it was expected that water particles lost velocity magnitude with increasing distance away from a rotating plate. For blades 2 and 3, the velocity of water particles above the blade faces was decreasing with height as expected. Blade 3 showed a much greater decrease as compared to blade 2. Figure 10 demonstrated that PIV measurements and ANSYS-Fluent predictions of the water particles velocities above all the blade faces showed agreement. Both CFD turbulence models showed almost the same variation; however, IDDES was more closely matching with the PIV measurements.

### Slippage factor k

The power input imparted by horizontal paddles of a flocculator to the water body is given by Eq. (1). Since the velocity of water is assumed to be less than the velocity of paddles by the factor **k**, therefore the relative velocity is represented as ${\text{(1 - }{\text{k}}\text{)}{\text{v}}}_{\text{p}}$ where ${\text{v}}_{\text{p}}$ is the velocity of paddles.

The validated CFD turbulent models were considered to determine the slippage factor **k** for the laboratory scale paddle flocculator. The power imparted to water by the mixer was needed to calculate the value of **k**. The torque of the mixer was calculated by exporting the torque coefficient ${\text{C}}_{\text{m}}$ from ANSYS-Fluent, and the power imparted to water was consequently computed by incorporating the rotational speed. Finally, the slippage factor was determined and presented in Table 2.

**Table 2:** Computation of the slippage factor **k** values.

Rotational speed	CFD model	${\text{C}}_{\text{m}}$ (unitless)	T (N-m)	P (watts)	k (unitless)
N = 3 rpm	SST k–ω	1.30	0.80	0.25	0.19
	IDDES	1.35	0.83	0.26	0.18
N = 4 rpm	SST k–ω	2.35	1.44	0.60	0.19
	IDDES	2.42	1.48	0.62	0.18

The results of Table 2 showed that each CFD turbulence model produced the same value of **k** for each of the two rotational speeds. The value of **k** from the SST k–ω model is slightly higher than that obtained by IDDES. Regardless of the variation, a general value for **k** could be considered as 0.18 for low rotational speeds such as 3 rpm and 4 rpm.

Ambiguity related to the slippage factor was noted in most designs. The outcome of this study showed that **k** was equal to 0.18 for low rotational speeds of 3 rpm and 4 rpm. The quantified value of **k** from this study could be used as an additional guideline in the design of paddle flocculators.

If the determined value of k of 0.18 is used, then the actual input power is higher by about 27%, which corresponds to 30%. Replacing this increase in the velocity gradient (G) equation and keeping the other parameters the same, one can find that G is actually increased by about 14%. This means that more mixing is provided than expected, which could be a positive indication from the point of view of the process. However, a higher G may lead to the breakage of the formed flocs. In order to preserve the flocs and keep them agglomerating, the actual input power has to be decreased for the same flocculation efficiency. This means less energy is input and thus savings in the cost of energy in the flocculation process is achieved.

## Conclusions

In this study, flocculation hydrodynamics were evaluated by investigating the velocity field of turbulent flow experimentally and numerically in a laboratory scale paddle flocculator. PIV experiments were conducted on the flocculator and the CFD ANSYS-Fluent was used to model the rotational flow inside the paddle flocculator. In both cases, time-averaged velocity contours of the water particles revealing the velocities surrounding the blades were developed. Two turbulence models were adopted; namely, the SST k–ω and the IDDES. Results showed that IDDES provided very slight improvement as compared to SST k–ω, yielding the latter sufficient in accurately simulating the flow inside the flocculator.

A Goodness-of-Fit Evaluation was adopted to study the convergence between PIV and CFD results, and to compare the results among the CFD turbulence models themselves. Furthermore, the velocity of water particles was plotted against the height above blade face starting from the origin which was at the face of the blade and upwards up to Y = 10 cm. Regarding blade 1 (inner), the velocity of water particles increased with height above the blade face for both rotational speeds. In general, this was unpredictable since it was expected that water particles lose velocity magnitude with increasing distance away from a rotating plate. For blades 2 (middle) and blade 3 (outer), the velocity of water particles above the blade faces was decreasing with height as expected. Blade 3 showed a much greater decrease as compared to blade 2. Furthermore, PIV and ANSYS-Fluent showed a very close variation for the velocity of water particles above all the blade faces. Both CFD turbulence models showed almost the same variation, where IDDES was more closely matching with the PIV results.

The study also focused on the quantification of the slippage factor **k**. The relationship between the input power imparted by the flocculator paddle blades to the fluid and (1 − **k**) is cubical. The literature does not specify exact values for **k**, but rather ranges are recommended. The quantitative determination of the slippage factor **k** of 0.18 in this study at low rotational speeds of 3 rpm and 4 rpm, whereas the conventional typical value is around 0.25. A lower **k** implies that the flocculator is achieving higher than expected velocity gradients, and thus more power, for the same rotational speed. Therefore, assuming all parameters the same, a reduction of k from 0.25 to 0.18 yields around 27–30% increase in the power imparted to the fluid, which results in increasing the velocity gradient (G) by about 14%. This means that more mixing is provided than expected, which could be a positive indication from the point of view of the process. However, a higher G may lead to the breakage of the formed flocs. In order to preserve the flocs and keep them agglomerating, the actual input power has to be decreased for the same flocculation efficiency. This means less energy is input and thus savings in the cost of energy in the flocculation process is achieved.

This work clearly demonstrates the benefits gained from using PIV and CFD in analyzing the paddle flocculator. Furthermore, the electric consumption for the flocculation unit at a drinking water treatment plant could be potentially decreased, and thus the most effective design is achieved.

## Supplementary Information

Supplementary Figures.

## Data Availability

The datasets used and/or analyzed during the current study are available from the corresponding author on reasonable request.
