# Supplementary material for: PIV and CFD investigation of paddle flocculation hydrodynamics at low rotational speeds
Source: Sci Rep. 2022 Nov 17;12:19742. doi: 10.1038/s41598-022-23935-x (PMC9672036; doi:10.1038/s41598-022-23935-x)
Supplement: Supplementary file 1 — Supplementary Figures. [file 41598_2022_23935_MOESM1_ESM.pdf]

Appendix A. Supplementary Data

PIV and CFD Investigation of Paddle Flocculation Hydrodynamics at Low Rotational Speeds

Jean George Chatila*[1], and Hrair Razmig Danageuzian[1].

[1] Department of Civil and Environmental Engineering, Lebanese American University, 309

Bassil Building, Byblos, Lebanon.

*Corresponding author:
Jean George Chatila
Department of Civil Engineering
Lebanese American University
P.O.Box. 36 Byblos, Lebanon
Phone: +961 3 643 462
Fax: +961 9 547 262
E-mail: jchatila@lau.edu.lb

Velocity profiles were measured and plotted at the face of each of the blades and at successive distances of 2.5 cm vertically upwards up to a height of 10 cm as seen schematically in Figure S1.

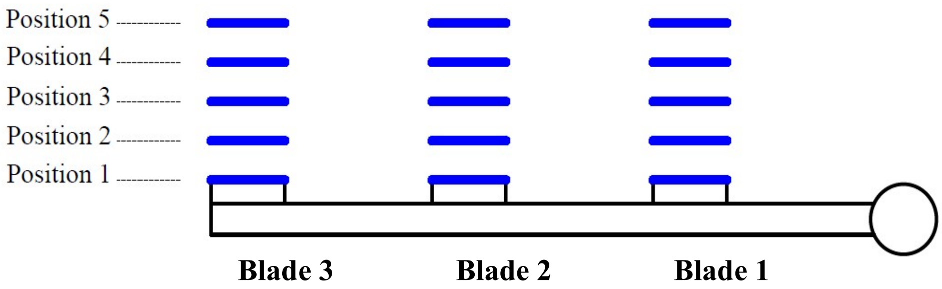

Figure S1 Positions of measured velocities at the face of each of the blades and at successive distances of 2.5 cm vertically upwards up to a height of 10 cm.

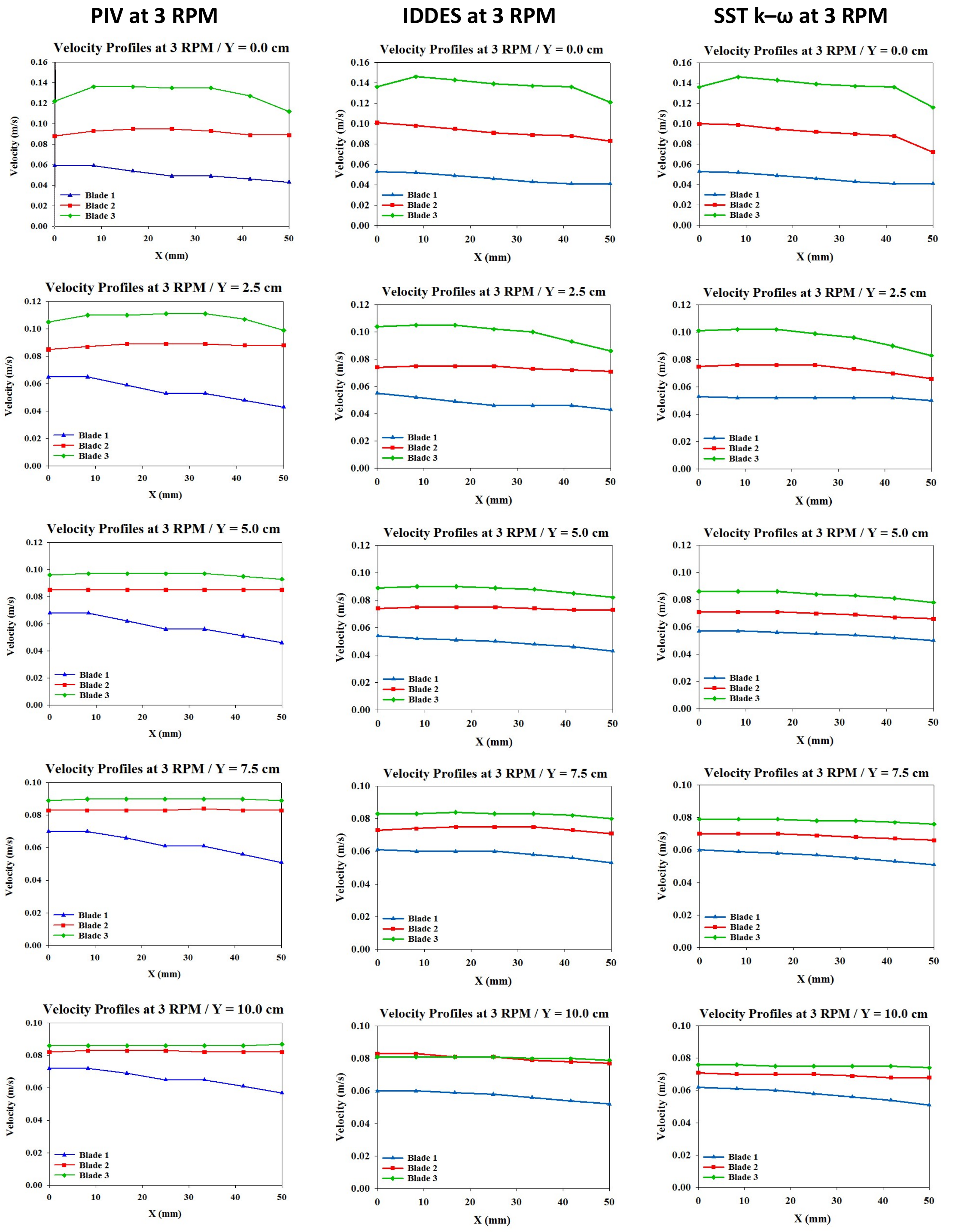

Figure S2 PIV, IDDES, and SST k–ω Velocity Profiles positioned at the face of the blades at horizontal increments of 10 mm up to blade width of 50 mm, and at vertical increments of 2.5 cm up to a distance of 10 cm above the blade at a rotational speed of 3 rpm.

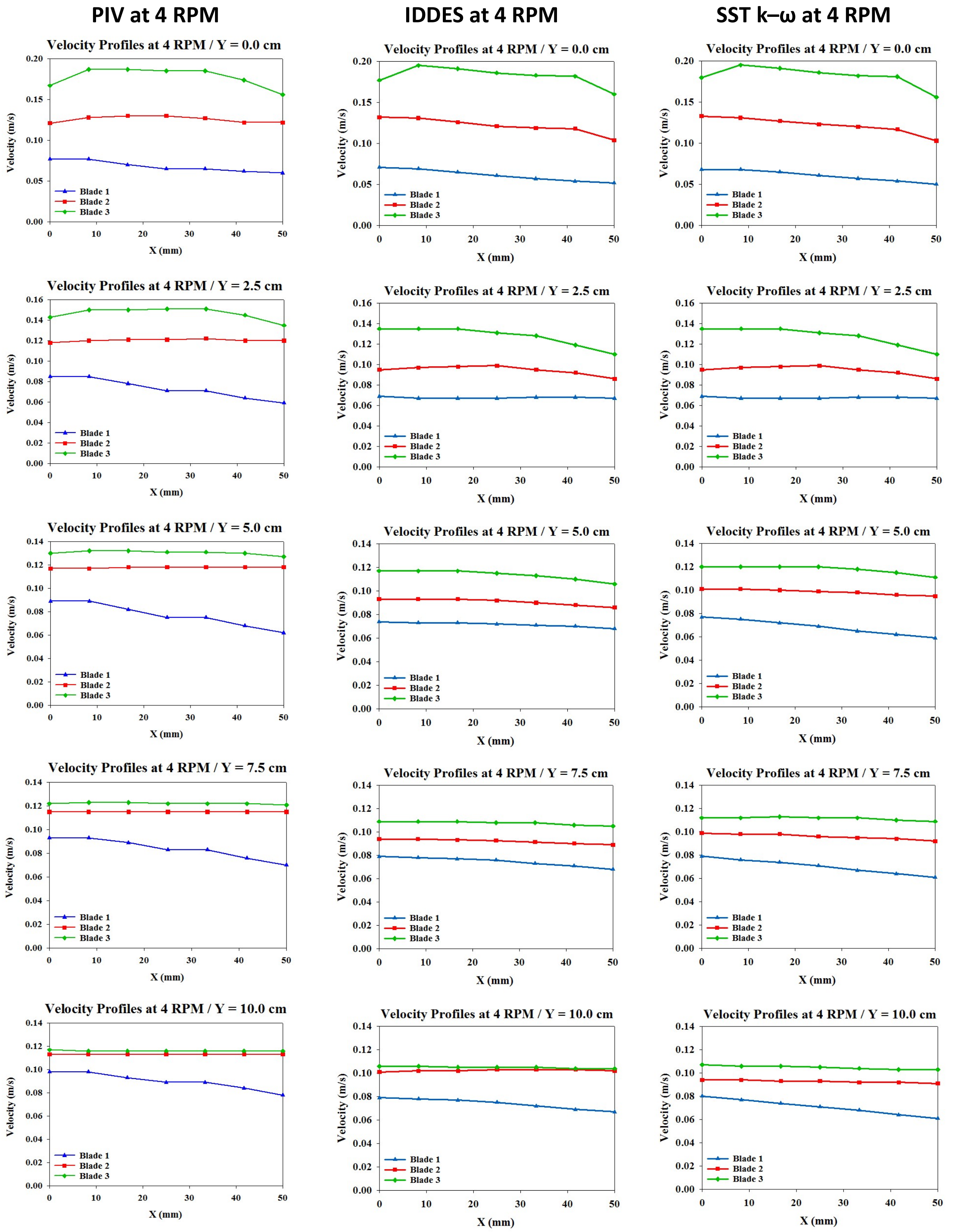

Figure S3 PIV, IDDES, and SST k–ω Velocity Profiles positioned at the face of the blades at horizontal increments of 10 mm up to blade width of 50 mm, and at vertical increments of 2.5 cm and up to a distance of 10 cm above the blade at a rotational speed of 4 rpm.
